# Quantifying the Impact of Mosquitoes on Quality of Life and Enjoyment of Yard and Porch Activities in New Jersey

**DOI:** 10.1371/journal.pone.0089221

**Published:** 2014-03-06

**Authors:** Yara A. Halasa, Donald S. Shepard, Dina M. Fonseca, Ary Farajollahi, Sean Healy, Randy Gaugler, Kristen Bartlett-Healy, Daniel A. Strickman, Gary G. Clark

**Affiliations:** 1 The Heller School for Social Policy and Management, Brandeis University, Waltham, Massachusetts, United States of America; 2 Center for Vector Biology, Rutgers University, New Brunswick, New Jersey, United States of America; 3 Mercer County Mosquito Control, Transportation and Infrastructure Department, Mercer County, West Trenton, New Jersey, United States of America; 4 Monmouth County Mosquito Extermination Commission, Monmouth County, Eatontown, New Jersey, United States of America; 5 Agriculture Research Service, United States Department of Agriculture, Beltsville, Maryland, United States of America; 6 Agriculture Research Service, United States Department of Agriculture, Gainesville, Florida, United States of America; 7 Agricultural Center, Louisiana State University, Baton Rouge, Louisiana, United States of America; University of Westminster, United Kingdom

## Abstract

The recent expansion of *Aedes albopictus*, a day-biting mosquito, to densely inhabited areas in the northeastern Atlantic states of the USA has dramatically increased the problem that mosquitoes create for urban and suburban residents. We quantified the impact of mosquitoes on residents' quality of life within the context of a comprehensive area-wide integrated pest management program to control *Ae. albopictus* in two counties (Mercer and Monmouth) in New Jersey. We interviewed residents of 121 randomly selected households in both counties between October and November 2010. We asked residents about their experience with mosquitoes in their neighborhood and the importance of the ability to relax outdoors without mosquitoes compared to other neighborhood characteristics (1 = not important, 5 = extremely important). We rated residents' utility based on paired comparisons to known states from the EuroQol health description system. The majority (54.6%) of respondents considered mosquitoes to be a problem. Respondents reported an average of 7.1 mosquito bites in a typical week during that summer. Mosquitoes prevented 59.5% of residents from enjoying their outdoor activities at least to some extent. Residents rated the mosquito acceptability (mean ± standard deviation) during that summer on a scale of 0 (mosquito invasion) to 100 (no mosquitoes) at 56.7±28.7, and their overall utility at 0.87±0.03. This is comparable to living with up to two risk factors for diabetes (i.e., abdominal obesity, body mass index of 28 or more, reported cholesterol problems, diagnosis of hypertension, or history of cardiovascular disease) or women experiencing menstrual disorders. Respondents rated the importance of enjoying outdoor activities without mosquitoes (4.69±0.80) comparable to that of neighborhood safety (4.74±0.80) and higher than that of a clean neighborhood (4.59±0.94). In conclusion, New Jersey residents reported that mosquitoes decreased their utility by 0.13, comparable to the loss from worrisome health risk factors, underscoring the importance of controlling this problem.

## Introduction

The day-biting mosquito *Aedes (Stegomyia) albopictus* (Skuse) became established in the United States in 1985, appeared in New Jersey in 1995, and is now the primary cause of service requests to local and state mosquito control programs [1]. While the disease risks associated with *Ae. albopictus* and other mosquito species have been extensively studied [2]–[5], the impact of mosquitoes on residents' quality of life, daily choices, behaviors, and use of resources has been rarely investigated [6], [7]. The World Health Organization defined health as “*a state of complete physical, mental and social well-being and not merely the absence of disease or infirmity* [8].” Understanding what factors influence residents' quality of life is important to guide public policy and assist policy makers in planning and allocating scarce public resources in a manner that enhances societal progress and enables the society to function efficiently and smoothly [9].

*Aedes albopictus*, commonly known as the Asian tiger mosquito, is a major concern for public health and mosquito control officials throughout its invasive range [10]–[12], as it continues to spread to new areas with high human population density [1], [4], [7], [11]. This species is highly adapted to urban and suburban areas, and commonly oviposits eggs in artificial containers [3], [13], [14]. *Aedes albopictus* is a vector of at least 22 arboviral diseases including dengue, chikungunya, West Nile virus, and yellow fever [15], [16]. With the increasing number of travelers to endemic countries diagnosed with arboviral infections, the presence of this species escalates the risk of local transmission of arboviral diseases throughout its range [2], [17], [18], as observed through autochthonous transmission of dengue and chikungunya in Hawaii, USA [19], France [20], Croatia [21], and Italy [22].

In southeastern Virginia, USA, the nuisance associated with *Ae. albopictus* affected the daily activities of 46.6% of residents, and forced 81.3% of residents to stay indoors due to mosquito bites [7]. In a study in Upper Rhine Valley, Germany, residents' preference for a mosquito control program targeting mosquitoes' nuisance was compared to mosquito control programs with potential economic benefits, such as promotion of gastronomy or tourism, it was found that residents were willing to pay 3.8 times the government's actual cost to control mosquito nuisance [23]. A Wisconsin, USA, study concluded that residents might be willing to pay on average $147 per household per year to reduce mosquito nuisance, compared to only $21 for programs targeting disease transmitting mosquitoes [24]. These two studies illustrate that the reduction of this nuisance was perceived as more valuable than controlling a disease threat or improving the economy.

The expansion of *Ae. albopictus* has been associated with a decline in native mosquitoes, e.g. *Ae. Triseriatus*, in urban areas of New Jersey and *Ae. aegypti* in Florida and other parts of the southeastern United States [25], [26], and a doubling in the share of residents' mosquito complaints due to *Ae. Albopictus* and an overall increase in service requests in New Jersey [1], [27]. As part of a comprehensive area-wide integrated pest management project to control *Ae. albopictus* [28], [29], this paper describes the experience of Mercer and Monmouth Counties residents' with mosquitoes, quantifies the impact of mosquitoes on their quality of life in terms of utility scores, and estimates the maximum amount they are willing to pay for one additional imaginary work-free and mosquito-free hour spent in yard and porch activities. These results will inform cost-effectiveness and benefit-cost studies when considering the contribution of mosquito control programs in reducing nuisance as well as potential risk of diseases. The findings should help guide public policy to define priority areas and help allocate scarce public resources in the most efficient way to address residents' preferences [9].

## Methods

### Overview

We aimed to quantify the benefits (in monetary value) and effectiveness (in utility value) of an area-wide integrated pest management (AW-IPM) project to control *Ae. albopictus*, implemented in Mercer and Monmouth Counties in New Jersey, from the residents' perspective. The AW-IPM project helped residents to use their yards and porches and enjoy activities there without the nuisance of mosquitoes. We selected three measures for this evaluation. The first was the reduction in the average number of hours lost during a typical summer week engaged in yard and porch activities–eating and cooking in yard or porch, gardening, relaxing and socializing, playing, and maintaining house or care–due to mosquitoes. Annual mailed surveys conducted from 2008 through 2011 in the study sites, with sample sizes ranging from 310 in 2008 to 548 in 2011, allowed us to measure the effectiveness of the AW-IPM project in reducing the number of hours lost per yard and porch activity due to mosquitoes [30]. The second was monetary value of this reduction in hours lost, derived by valuing each hour gained using the contingency valuation method. The third selected measure is the improvement in residents' utility, or satisfaction. We estimated the utility associated with mosquitoes using three methods: (1) the visual analogue scale, (2) a state tradeoff (adapted from the time tradeoff method) that compares experiencing an average day with mosquitoes as they were the summer of 2010 in the respondent's yard and porch with selected health states, and (3) a disease tradeoff based on five diseases with mild disability weights derived from the Global Burden of Disease studies [31]. The utility score range lies between two values: 1 denotes perfect health and 0 denotes deteriorated health status similar to or equal to death, therefore, a higher utility score indicates higher wellness or satisfaction. The utility score will be used to evaluate the effectiveness of the AW-IPM project in improving residents' utility or level of satisfaction, calculate Quality Adjusted Life Years or QALYs gained, and perform a cost-effectiveness analysis.

### Study design

To quantify the hours gained and to measure the utility associated with mosquitoes we selected for potential face-to-face interviews a random sample of 400 households, a subset of the larger mailed household survey conducted in 2010 [28]. The mailed household survey aimed to document the change in the number of hours residents spent engaged in yard and porch activities due to mosquitoes, mosquito-control expenditures, knowledge of mosquito-control measures, and action taken to control mosquitoes, in addition to demographic characteristics [27].

The interviews were conducted between the first week of October and the first week of November 2010, by six trained, two-student teams from the Department of Entomology at Rutgers University: one student interacted with the interviewee, while the other student documented responses and provided visual aid materials when needed. The interviews were conducted between 10am and 8pm, with an average 50 minutes per interview. Three attempts were made to contact each selected household.

### Ethics statement

The investigators sent selected residents a letter a week in advance about the study providing the objectives, approach (interview at their door or home), and contacts for the investigators and Brandeis University Institutional Review Board (IRB) in case of any concerns. The protocol entailed oral consent as the study involved only spoken, private responses to questions that were considered neither risky nor sensitive. Subjects were compensated $10 in appreciation for their time. Consent was documented on study forms by the interviewer's name and date of the interview. The IRB at Brandeis University reviewed and approved the research protocol (IRB number: 09012).

### Approaches to quantify mosquito impact

#### Visual analogue scale (VAS) valuation

To estimate the unpleasantness associated with mosquito abundance, we first used VAS as a rating scale to derive preference weights and create an interval scale [32]. We asked residents to rate the mosquito acceptability during a typical 2010-summer week on a scale from 100 (referring to no mosquitoes–best scenario) to 0 (referring to an invasion of mosquitoes–worst scenario).

#### EuroQol states trade-off (EuroQol-STO)

Time-Trade- off (TTO) is a tool used in health economics to determine the quality of life of a patient or group. This tool instructs individuals to choose between living a fixed number of years (usually 10 years or F) in a specified health condition, to living Z years in perfect health. The difference Y, where Y = F–Z, denotes the number of years the respondent is willing to trade to move from living in the specified health condition to living in perfect health. The number of years of perfect health selected (Z) is then converted into a utility score (generally Z/F) and used to calculate QALYs [33].

We modified the TTO method to derive the mosquito-abundance-utility score by allowing residents to elicit preferences between alternative health states, instead of time, and living an average day with mosquitoes. We first asked respondents to remember how it was living an average day with mosquitoes as they were in their yard and porch that summer. We then asked them to select which is a worse state in their opinion: living an average day with mosquitoes as they were in their yard and porch that summer or living in each of five health states selected, as presented in Survey S1. We conceptualized these states as rungs on a ladder, so the respondent could indicate the rung below which their mosquito acceptability fell. These five health states were derived from EuroQol EQ-5D descriptive system, which compromise health dimensions of mobility, self-care, usual activities, pain/discomfort, and anxiety or depression. Each dimension has three levels: no problems, some problems, and extreme problems. These dimensions were analyzed to generate a utility score that ranges between 1 (denoting perfect health) and 0 (equal to death). The five selected health states had utility scores ranging from 0.897 to 0.806, the range that we expected would apply to most respondents [34].

#### Disease states trade-off (D-STO)

Using paired comparison questions and population health equivalence, the Global Burden of Disease 2010 study quantified disability weights associated with one year in each specified health condition, where 1 implies a health loss equivalent to death and 0 implies no loss of health or perfect health [31]. These weights, which are the reverse of weights in QALYs, are used to compute the disability-adjusted life-years (DALYs) reported in burden of disease studies. Similar to EuroQol-STO, we selected five diseases with mild disability weights per unit time (shown in parentheses): influenza (0.210), stomach flu (0.281), severe hearing loss (0.032), wrist fracture (0.065), and bronchitis (0.210) [31]. Again, we asked respondents to recall living an average day with mosquitoes as they were in their yard and porch that summer. We then asked them to select which is a better state in their opinion: living an average day with mosquitoes as they were in their yard and porch that summer or living an average day with each of these five health conditions.

#### Contingent valuation

To estimate the benefit associated with a program that reduces mosquitoes' nuisance, we asked respondents to rank five porch and yard activities (i.e., eating and cooking outside, playing, relaxing and socializing, gardening, and maintaining their car or house), and to state the maximum amount they were willing to pay for one additional hour engaged in each of these activities with reduced mosquito nuisance. We started the bid with $1. Four cases (3.3% of our sample) reported extreme values (over $100 per porch or yard activity). We adjusted for these extreme values by winsorizing willingness to pay (WTP) values to the variable's 95th percentiles.

### The survey instrument

To measure the impact of mosquitoes on residents' quality of life we developed a four-section structured questionnaire to complement the mailed survey conducted annually from 2008–2011. The first section focused on interviewee's experience with mosquito bites in their neighborhood during a typical 2010 summer week, whether they were treated for bites, and, if so, the cost of treatment. The second section rated the importance of the ability to relax outdoors without mosquitoes compared to other neighborhood characteristics (1 = not important, 5 = extremely important), and the unpleasantness associated with mosquito bites compared to other unpleasant events that can occur in a typical neighborhood (1 = not unpleasant, 5 = extremely unpleasant). In the third section interviewees were asked to rate and rank the enjoyment associated with five porch and yard activities and to indicate their willingness to pay for one additional imaginary work-free mosquito-free hour each summer week engaged in each of five porch or yard activities. In the fourth section interviewees rated their current mosquito acceptability on a utility scale (similar to EQ-5D-VAS-visual analogue scale) from 100 (no mosquitoes) to 0 (mosquito invasion), and answered the EuroQol-STO and D-STO questions.

### Data analysis

Graduate students from Brandeis University coded the survey responses and entered the data into Excel spreadsheets (Microsoft Corporation, Redmond, WA). Twenty percent of the sample was reentered to check for consistency and quality of the data entry. Data were then transferred to STATA (College Station, TX) for analysis. Results are reported as unweighted means, standard deviations, and standard error of the means for continuous variables and frequencies for categorical variables. Then *t*-tests and Chi-square tests were performed for hypothesis testing.

To estimate each respondent's EuroQol-STO mosquito-abundance utility score, we first coded as 1 those items respondents reported as mosquitoes were worse than the comparison states, and coded items not worse as 0. We then defined a 1-based (worst condition) and 0-based (best condition) utility for each respondent. The 1-based utility was based on items coded as 1;; i.e., those EuroQol descriptive health states for which living an average summer day with the then level of mosquitoes was considered to be worse. The lowest utility of those descriptive states was the 1-based utility score. The 0-based score was the items coded as 0; i.e., the utility of the EuroQol descriptive health state with the highest utility for which the respondent stated that living an average summer day with the then level of mosquitoes was not considered worse.

If mosquitoes were worse than all five EuroQol descriptive health states, we set both the 1-based and 0-based utility scores at 0.806, a value extrapolated downward from the utilities of the five health states above. If mosquitoes were equal to or better than all EuroQol descriptive health states, we set both the 1-based and 0-based utility scores at 0.897, a value extrapolated upward from the utilities of the five states below. Finally, we set each respondent's EuroQol-STO utility as the average of their 0-based and 1-based utilities. For respondents whose answers were consistent with the Euro-Qol ordering of health states, their 0-based and 1-based utilities were identical.

To derive the D-STO utility we used similar categories to those used to derive the EuroQol-STO, with minor modifications. For category 1, we defined a better disease state as the one just better than the mildest of these diseases, which was intellectual disability, mild (disability weight 0.031). For category 2, we defined a worse disease state as the one just worse than the most severe of these diseases, which was neck pain (disability weight 0.286). To help readers interpret the resulting utility values, we identified health states in the Global Burden of Disease Study with comparable utility values [31], [35], [36].

To validate the WTP results, we estimated a logit model about whether the respondent was willing to pay some finite amount to avoid mosquitoes as a function of household characteristics, exposure to mosquitoes and cost associated with mosquitoes. Additionally, we modeled the positive maximum amount residents were willing to pay for each of the selected yard and porch activities on household characteristics, exposure to mosquitoes, and cost associated with mosquito control and mosquito-related healthcare services using log-linear regression models.

## Results

### Household characteristics

Of the 400 randomly selected addresses, 121 households completed the interview, 58 households were not interested in participating in the study, 9 addresses were not residential units, and 6 addresses were outside the study areas. The remaining 206 households were not successfully contacted. Of the 385 valid addresses, the response rate was 31.4% (121/385), and the cooperation rate was 67.5% (121/(121+58)).

The majority of respondents (55%) were from Monmouth County, women (62%), and in the labor force (57%). Table 1 compares the main characteristics of the study sample with those in the AW-IPM project's selected sites and counties. While some of the variables showed statistically significant differences from the population, the excess of females and larger households is consistent with the study procedures.

**Table 1:** Household characteristics of respondents compared to study sites and counties, 2010.

Variable	Study sites	Study sample	Sig.
Number of households in county (N = 121)†			***
Monmouth	33%	55%	
Mercer	67%	45%	
Child at home+ (N = 121)			NS
Household with one or more people under 18 years	35%	37%	
Respondent's gender (N = 121)			*
Female	51%	62%	
Respondent's age (N = 88)			***
35–44	19%	19%	
45–54	19%	33%	
55–64	21%	27%	
65–74	23%	10%	
75 and up	18%	10%	
Respondent's level of education# (N = 107)			NS
Less than 9th grade	7%	6%	
9–12 grade	9%	7%	
High school graduate	34%	42%	
Some college no degree	19%	23%	
Associates degree	8%	4%	
Bachelor degree	16%	11%	
Graduate or professional	8%	7%	
Average household size (N = 121)	2.69	3.20	***
Respondent's employment status (N = 120)			***
In the labor force	60%	57%	
Unemployed looking for a job	7%	0%	
Not in labor force	33%	43%	

† N denotes the number of respondents to the question.

+ child under 18.

# population 25 years and over.

*p<0.05;

*** p<0.001 based on Chi Squared test (for discrete variables) and t-test (for continuous variables); NS = Not statistically significant.

### Experience and expenditure associated with mosquito bites

The majority (54.6%) of respondents considered mosquitoes to be a problem, with 30.6% rating mosquitoes as a moderate problem, 12.4% as a severe one, and 11.6% as an extremely horrible one. Mosquitoes prevented 59.5% of respondents from enjoying their outdoor recreational activities, at least to some extent. During a typical summer week, 80.2% of respondents reported being bitten at least once; 77.7% were bitten while outdoors and 23.1% were bitten while indoors. Overall, respondents experienced an average (±standard error of the mean, SEM) of 7.1±1.1 mosquito bites per week. Respondents reported bites at all times of the day or night, including the daytime, when the Asian tiger mosquito bites. The distribution of times was: early morning (11.6%), late morning (11.6%), late afternoon (30.6%), early evening (52.1%), and night (31.4%). These percentages sum to more than 100%, as residents reported being bitten during multiple periods in the day. Of those bitten, 49.6% used existing products at home to treat their bites, 34.7% bought new products, and 4.2% saw a health care provider to treat their bites (1.7% a specialized doctor, and 2.5% a nurse or primary healthcare doctor). For all those interviewed, the average (±SEM) amount paid per person on itching and mosquito bite treatment was $9.14 (±$1.98), on medical providers $9.71 (±9.19), while their insurance coverage paid on average $13.14 (±3.91). The respondents' medical cost associated with relief and treatment of mosquito bites for the study areas during the summer period (assuming summer is 13 weeks) averaged across all residents, including those with no expenditure, $31.99 (±10.57) per resident, of which 28.5% was paid by households for itching and mosquito bite treatment/products, 30.4% paid by households as co-payment for health consultancies, and 41.1% by insurance companies for medical fees. This distribution shows that multiple sectors were affected financially by mosquito bites. We found no statistically significant difference by gender, except for number of residents who reported being bitten: men were more likely to be bitten compared to women, and women were more likely to use or buy a product to treat bites compared to men. We found no significant difference by county, except for the overall level of rating of the mosquito problem in the neighborhood.

### Importance of mosquito control compared to other public services

Respondents rated the importance of enjoying porch and yard outdoors activities without mosquitoes' nuisance (4.69) second to that of neighborhood safety (4.74) and higher than that of a clean neighborhood (4.58). As shown in Table 2, residents' experiencing 7 mosquito bites in a week was rated as the most unpleasant event (4.71) followed by having trash in their block (4.61), and having mosquitoes outside their residence (4.45).

**Table 2:** Respondents' perceived importance and unpleasantness of certain aspects of neighborhood, 2010.

Aspect of living in a neighborhood (N = 121)	Mean	SEM
Importance of ability to*		
Walk around your neighborhood without seeing garbage or litter	4.58	0.09
Walk in your neighborhood at night without fear of crime	4.74	0.07
Use parks and playgrounds	4.26	0.11
Cross streets in your neighborhood safely	4.59	0.09
Relax, barbecue, play and socialize in your yard or porch without mosquitoes	4.69	0.07
Unpleasantness associated with+		
Having broken or missing street signs on your block	3.79	0.13
Having trash in your block	4.61	0.08
Seeing graffiti on lamppost or telephone pole on your block	4.21	0.11
Having mosquitoes outside your house	4.45	0.10
Getting seven mosquito bites in a week	4.71	0.07

*1 = not important, 5 = extremely important.

+ 1 = not unpleasant, 5 = extremely unpleasant.

Notation: SEM denotes standard error of the mean.

### Porch and yard activities: importance and willingness to pay

As presented in Table 3, the activity rated as most important was relaxing and socializing in the yard or porch (89.2%), followed by eating and cooking outside (82.7%). The order of these ratings paralleled that of their enjoyment. When asked for the maximum amount respondents were willing to pay for one additional imaginary work-free, mosquito-free hour per summer week engaged in these activities, the ranking of the average maximum amounts they were willing to pay was very similar to the enjoyability ranking. We found one exception in gardening ($7.74), which was ranked fourth but the amount was 6.6% higher than the amount they were willing to pay for playing in the yard ($7.26).

**Table 3:** Enjoyment associated with yard and porch activities and willingness to pay (WTP) for one additional work-free, mosquito-free hour per summer week engaged in each of these activities.

Yard and porch activity	Rate as very important or important	Ranking of activity by enjoyment*	Mean max. WTP	SEM max. WTP
Relaxing, socializing, talking, reading, etc.	89.2%	1	$10.75	1.39
Eating or cooking outside	82.7%	2	$10.43	1.36
Playing catch, Frisbee, bocce, etc.	64.4%	3	$7.26	1.04
Gardening	57.0%	4	$7.74	1.29
Maintaining house or car	53.7%	5	$6.47	1.05

* 1 Denotes highest ranked (most enjoyable) activity.

Notation: SEM denotes standard error of the mean.

Of those interviewed, 92.4% stated their willingness to pay for this imaginary hour engaged in at least one of these activities: 85.7% were willing to pay at least $0.25 to enjoy eating or cooking outside, 76.5% to play in yard or porch, and 75.6% to enjoy gardening. Eighty-nine percent were willing to pay at least $0.50 to relax and socialize, and 71.4% to maintain their house or car. Table 4 displays determinants of WTP from logit and log-linear regressions.

**Table 4:** Validation of willingness to pay (WTP).

Variables	Logit:		Regression on log of positive values of maximum WTP per hour for								
	WTP positive, Exp(B)		Eating		Playing		Relaxing		Gardening	Maintenance	
Household characteristics											
Resident of Monmouth county	0.376		−0.082		0.006		−0.053		0.029	0.004	
Has one or more children less than 18	0.409		0.100		0.074		0.121		0.071	0.101	
Associate degree or higher	0.055	**	−0.014		−0.022		0.151		−0.080	−0.073	
Elderly	0.482		−0.022		−0.066		−0.056		0.079	−0.039	
Female	0.284		−0.128		−0.206	+	−0.184	+	−0.086	−0.14	
Employed full time	0.811		0.187		0.155		0.043		0.167	−0.009	
Number of people living in household	0.978		−0.054		0.046		−0.094		0.083	0.038	
Exposure to mosquitoes											
Hours they would have spent on yard and porch activities	1.009		0.000		−0.002		0.072		−0.057	0.055	
Perceived severity of mosquitoes (severe or horrible vs. somewhat or less)	1.926		−0.056		−0.070		−0.077		−0.126	−0.118	
Cost associated with mosquitoes ($ during summer)											
Mosquito control in household	1.037		0.133		0.129		0.159		0.158	0.146	
Medical expenditures for bites	1.149		0.215	+	0.208	+	0.208	+	0.157	0.296	*
Model summary											
Number of observations	119		102		91		106		90	85	
Log likelihood	44.158										
Chi squared	24.508	*									
Adjusted R-squared			0.034		0.024		0.05		−0.02	−0.003	

+ p< 0.1;

* p<0.05;

** p<0.01.

Willingness to pay any amount was positively associated with residency in Mercer County compared to Monmouth County, having at least one child under the age of 18 years of age, attaining some higher education, being female, being employed full time, being bothered by mosquitoes to the extent that one could not spend the time they desired engaged in yard and porch activities, and incurring some cost associated with mosquito control or health expenditure associated with mosquito bites. Health expenditures associated with mosquito bites showed positive statistically significant impacts on residents' WTP for this additional mosquito free hour (eating p = 0.051, playing p = 0.076. relaxing p = 0.051, maintenance = 0.018).

### Loss in utility due to mosquitoes

On average (±SD), residents rated their overall mosquito acceptability score during that summer on a scale of 100 (no mosquitoes) to 0 (mosquito invasion) at 56.74±28.73. Table 5 presents the percentages of respondents' stating that living an average day with mosquitoes in their yard and porch during the summer of 2010 was worse than living an average day with the specified comparator health conditions and diseases. The average (±SD) utility based on EuroQol-STO was 0.87±0.03, corresponding to a utility loss of 0.13. The average (±SD) utility based on the five diseases (D-STO) was 0.79±0.71, corresponding to a disability of 0.21±0.30, which is close to the disability weight attributed to moderate diarrhea (0.202) [31]. We found no significant difference by gender or county.

**Table 5:** Percentage of respondents rating an average day with mosquitoes during the summer of 2010 as worse than each comparator condition.

Comparator	Comparator utility score	Percent*
EuroQol health state descriptions for EuroQol-STO (n = 121)		
11211: some problem performing usual activities	0.888	41.32
21111: some problem walking around	0.880	33.06
11112: moderately anxious	0.876	26.45
22111: some problem walking around, and some problem with self-care	0.823	30.58
12112: some problem with self-care and moderately anxious	0.815	21.49
Disease states for D-STO (n = 120)		
Severe hearing loss	0.968	11.57
Wrist fracture	0.935	14.05
Influenza	0.790	13.22
Bronchitis	0.790	8.26
Stomach Flu	0.719	09.92

* As items were independent the percentages could sum to more or less than 100%.

### Consistency checks

The average (±SD) overall self-rated mosquito acceptability score (using VAS) was significantly lower for respondents with a moderate to severe mosquito acceptability compared with those who stated mosquitoes as a mild or no problem in their neighborhood (47.67±26.08 and 67.62±28.18, respectively; t(119) = 4.04 p<0.001). The pattern of the EuroQol-STO utility derived for EQ-5D-3L was similar to that observed for VAS scores; the average (±SD) EuroQol-STO utility was significantly lower for respondents with a moderate to severe mosquito acceptability compared with those who stated mosquitoes as a mild or no problem in their neighborhood (0.86±0.03 and 0.87±0.03, respectively; t(119) = 2.08 p = 0.04. The two variables are significantly correlated, *r* (119) = 0.29, p<0.01.

The pattern of the D-STO utility index derived for disability weights was different from that observed in the VAS scores and the EuroQol-STO utility derived from EQ-5D; the average (±SD) indices were significantly higher for respondents with a moderate to severe mosquito experience compared with those who stated mosquitoes to be a mild or no problem in their neighborhood (0.80±0.04 and 0.79±0.02, respectively; t(118) = −2.39 p = 0.02). We found no correlation between the utility score derived from the EuroQol EQ-5D-3L utility score and the utility obtained from the five disability weights; χ^2^(65) = 67.63, p = 0.39.

## Discussion

Our study has investigated and explored the impact of mosquito abundance on residents' life in two counties in New Jersey. Our results support observations from previous studies, which indicated the high value residents place on reduction of mosquito nuisance. We also measured the maximum amount they are willing to pay for an active program that can significantly reduce mosquito nuisance and improve the quality of their local environment [7], [23], [24]. Our results show that on average (±SD) residents were willing to pay the amount of $8.53 (±12.45) per person per week for one additional mosquito-free hour each summer week spent engaged in any yard or porch activity or $9.48 (±13.05) per person per week for an additional recreational mosquito-free hour.

Additionally, our study shows that mosquitoes are a major concern for residents: mosquitoes are forcing them to sacrifice some of the time they would have ideally spent outdoors engaged in yard or porch activities. The high percentage of respondents (80%) being bitten, and the fact that the majority of these bites took place outdoors, resulted in less time spent outdoors. This high rate of respondents' reporting being bitten at least once during a summer week should also be cause for concern about the rapid spread of arboviral diseases, including chikungunya virus, should it ever be introduced in the United States. In Réunion and nearby islands during the 2004–2007 epidemics, much of the population was infected with chikungunya virus within a few months [37].

The mailed survey results show that respondents lost on average (±SEM) 8.43±1.07 hours during a typical 2010 summer week due to mosquitoes, of which 2.42±0.39 hours were lost on average from less eating and cooking outdoors, and 2.81±0.43 hours from less relaxing and socializing. Assuming a constant marginal utility for each additional hour spent outdoors and no budget constraints, our results suggest that residents were willing to pay $71.91±76.94 per person per summer week, or $934.80±1,000.24 per 13-week summer to enjoy their yard activities without mosquitoes.

The negative coefficients associated with the maximum amount residents were willing to pay for one additional imaginary work-free/mosquito-free hour for all activities except relaxing and respondents' education agreed with previous research that found higher education was associated with lower willingness to contribute to mosquito control [38]. Likewise, we found that, on average, more highly educated respondents spent less time in yard and porch activities and had lower WTP. Similarly, the higher WTP in Mercer County is consistent with its 13% lower median household income in 2008–2012 [39], [40].

Our study is the first, to our knowledge, to quantify the impact of mosquito abundance and nuisance on residents' quality of life. We used three approaches: the VAS, based on the theory of measurable multi-attribute value function used to order differences in individuals' preferences between alternatives [41], gave an overall self-rated mosquito acceptability status of 56.74. We used EuroQol EQ-5D-3L descriptive system to derive the EuroQol-STO utility score of 0.87, a utility comparable to living with up to two risk factors for diabetes (i.e., abdominal obesity, Body Mass Index of 28 or more, reported cholesterol problems, diagnosis of hypertension, and history of cardiovascular disease) or women experiencing menstrual disorders [35], [36]. In the third approach we used the D-STO to derive the mosquito nuisance disability weight of 0.21, comparable to a severe episode of influenza [31].

As expected, the overall scores derived from the EQ-5D-3L, whether measured by VAS or utility index score, were significantly higher in cases where the mosquito experience in the neighborhood was moderate, severe or horrible as stated by respondents, compared to cases where respondents faced no problem or only a mild problem with mosquitoes. However, the D-STO utility scores obtained by comparing the mosquito acceptability that summer with a specified disease were unexpected and disagreed with the results obtained from the EuroQol-STO utility, showing a higher utility score for cases with moderate, severe or horrible mxosquito experience in the neighborhood as stated by respondents compared to those who experienced a mild or no mosquito problem. This might be due to two factors: the first is the low number of respondents who stated that mosquitoes are worse than a disease (average of 11% of respondents) compared to health states (average of 31% of respondents). The second factor is related to the question itself. The results suggest that residents faced difficulties in trading off a day living with mosquitoes to a day living with one of the selected diseases, which are associated with great discomfort and disability and might lead to confinement in bed. However, they were more open and willing to trade-off some moderate states that they can adjust to and live with.

Some limitations of our study must be acknowledged. First, the respondents may have been persons who cared more about mosquitoes than non-respondents. However, the favorable cooperation rate (67.5%), and general similarity on education between respondents and the study sites suggests any potential bias would be limited. Second, the survey assesses reported or intended actions, rather than objectively observed activities. However, the substantial internal consistency (e.g. the same activity–relaxing and socializing–was ranked highest on importance, enjoyability, and WTP) suggested that the responses were thoughtful. Third, to shorten the interviews, we used only selected items from the EQ-5D instead of the full instrument, but may have lost some precision. Fourth, our interviews occurred weeks after the peak biting season, so respondents may not have fully recalled the nuisance they experienced. Fifth, our method analyzed the mosquito nuisance but did not include the potential health threat associated with disease carrying mosquitoes. Incorporating this factor might have increased the utility loss further. Sixth, we presented the unweighted results, since we found no statistically significant differences by county or gender at the customary significance level of p<0.05. However, supplementary analysis showed differences between gender groups on the EuroQol-STO utility score at the borderline level of p = 0.06. As weighting the results changed only the third decimal place of our results (from 0.871 to 0.873) and lowered precision, we decided to report the unweighted results. Further research could extend this work by applying additional methods, such as a direct utility elicitation technique such as TTO or standard gamble.

Conversely, our investigation also has several strengths. Our sample of 121 cases is adequate to estimate the utility lost due to mosquitoes as it exceeds the suggested minimum of 100 respondents to valuate one condition [42]. To our knowledge, this is the first study to quantify the utility associated with mosquito abundance using three different methods, and it is the first study to put a value on an hour free of mosquitoes spent in yard or porch activities.

The present paper provides evidence of the impact of mosquitoes on residents' quality of life. New Jersey residents report a 0.13 decrement in utility due to mosquitoes, comparable to worrisome health risks. The mosquitoes' nuisance effect is further emphasized by the perceived importance respondents placed on mosquito control activities compared to other public services, such as access to public parks and trash collection.

## Supporting Information

Survey S1 (PDF)
