# Supplementary material for: Quantifying the Impact of Mosquitoes on Quality of Life and Enjoyment of Yard and Porch Activities in New Jersey
Source: PLoS One. 2014 Mar 6;9(3):e89221. doi: 10.1371/journal.pone.0089221 (PMC3945781; doi:10.1371/journal.pone.0089221)
Supplement: Survey S1 — (PDF) [file pone.0089221.s001.pdf]

New Jersey Area Wide Pest Management Project
Interview Tool
October 2010

Donald Shepard, Yara Halasa and Eve Wittenberg
Brandeis University

The impact of mosquitoes on residents is of great interest to citizens and policy makers in New Jersey because of a new mosquito species in the area called the Asian tiger mosquito. These mosquitoes are most active during the day and require small amounts of water to breed. I would like to ask you some questions about how mosquitoes affect your life, particularly yard and porch activities. This interview will take about 25 minutes. On completion, you will receive a payment of $10 as a token of appreciation for your time.

1. How has your experience been with mosquitoes in your neighborhood this summer? Would you consider it not a problem, a mild problem, a moderate problem, a severe problem, or an extremely horrible problem? (**NOTE: MARK ONE**)
    a. Not a problem
    b. Mild
    c. Moderate
    d. Severe
    e. Extremely horrible

2. Do you have a child in your household?
    a. Yes
    b. No…………………………………………………..Go to Question 5

*If you have only one child, consider him or her the “selected child.” If you have more than one child in your household, then questions asking about the “selected child” in your household refer to your oldest child in **elementary school**.

3. What is the age of your selected child?______ years **(NOTE: WRITE 1 IF 1 YEAR OR LESS)**

4. What grade is your selected child in? ________ **(NOTE: WRITE NONE IF NOT IN SCHOOL)**

5. During a typical week this summer, how many **hours per week** did you and/or your selected child spend on your porch, steps, or yard engaged in each of the following activities? **(NOTE: IF NONE, PUT 0)**

**Activity on outdoor porch or yard**	Hours **you** spent in a typical **week**	Hours **selected child** spent in a typical **week**
a. Eating or cooking out		
b. Gardening or mowing lawn		
c. Maintaining house or car		
d. Playing catch, frisbee, bocce, horseshoes, croquet, volleyball/badminton etc.		
e. Relaxing, socializing, talking, reading, hanging out, smoking		
f. Others– please specify below:		

6. During a typical week this summer, to what extent did mosquitoes prevent **you** from enjoying outdoor recreational activities? (**NOTE: MARK ONE**)

**Not at all**	**A little bit**	**Somewhat**	**Very much**

7. During a typical week this summer, to what extent did mosquitoes prevent **your selected child** from enjoying outdoor recreational activities? (**NOTE: MARK ONE**)

**Not at all**	**A little bit**	**Somewhat**	**Very much**

8. We are interested in the impact of mosquitoes on outdoor porch or yard activities. During a typical week this summer, how many **hours per week would you and/or your selected child have spent** on your outdoor porch or yard if you and/or your selected child had no concerns over mosquitoes? **(NOTE: IF NONE, PUT 0)**

**Activity**	Hours **you** would have spent in a typical **week**	Hours **selected child** would have spent in a typical **week**
a. Eating or cooking out		
b. Gardening or mowing lawn		
c. Maintaining house or car		
d. Playing catch, frisbee, bocce, horseshoes, croquet, volleyball/badminton etc.		
e. Relaxing, socializing, talking, reading, hanging out, smoking		
f. Others– please specify below		

9. Thinking about a typical week over this past summer, were you bitten by mosquitoes at all? Was your selected child bitten by mosquitoes at all? If so, where were you and your selected child when they were bitten?

**Response**	**You**	**Selected child**
Yes, Outdoors		
Yes, Indoors		
Not bitten		
Don't know		

10. How many mosquito bites in total did you get during this typical week? ____

11. How many mosquito bites in total did your child get during this typical week?

____

12. In which time period(s) were you and/or your selected child bitten by a mosquito? (**NOTE: MARK ALL THAT APPLY**)

**Response**	**You**	**Selected child**
Early morning		
Late morning		
Afternoon		
Evening		
Night		
Not applicable or don't know		

13. Did you do any of the following for your bite or your child's bite during a typical summer week? (**NOTE: MARK ALL THAT APPLY**)

**Response**	**You**	**Selected child**
a. Treated at home (e.g. available drug, cream, spray you have at home)		
b. Bought product(s) (e.g. drug, cream, spray)		
c. Saw doctor or nurse		
d. Saw specialist doctor (e.g. dermatologist)		
e. Did not consult anyone and did nothing		
f. Others (specify)		

14. How much money did you pay out of your own pocket for this treatment or consultation? **(NOTE: IF NONE, PUT 0)**
    a. Purchased product $_____
    b. Doctor or nurse consultation $_____
    c. Specialized doctor (e.g. dermatologist) $______
    d. Others:____________________________________

15. I want you to think of the following aspects of your life in your neighborhood and how important or unimportant they are to you personally (**NOTE: READ THE LIST**). Now, I would like you to rate each one of these aspects according to how important it is for you. 1 is not at all important and 5 is extremely important) (**NOTE: HAND CARD Q15 TO INTERVIEWEE AND GIVE TIME TO THINK**)

→

Not important (1) **Extremely important (5)**

a. Ability to walk around your neighborhood without seeing garbage or litter	1	2	3	4	5
b. Ability to walk in your neighborhood at night without fear of crime	1	2	3	4	5
c. Ability to use parks and playgrounds	1	2	3	4	5
d. Ability to cross streets in your neighborhood safely	1	2	3	4	5
e. Ability to relax, barbecue, play and socialize in your yard or porch without mosquitoes	1	2	3	4	5

16. I want you to think of the following **unpleasant** events (**NOTE: READ THE LIST BELOW**). Now, I want you to rate them according to how unpleasant they are for you. Rate the unpleasantness from 1 (not unpleasant) to 5 (extremely unpleasant) (**NOTE: HAND CARD Q16 TO INTERVIEWEE AND GIVE TIME TO THINK**)

→

Not unpleasant (1) **Extremely unpleasant (5)**

a. Having broken or missing street signs on your block	1	2	3	4	5
b. Having trash on your block	1	2	3	4	5
c. Seeing a bicycle rider on your street	1	2	3	4	5
d. Seeing graffiti on lamppost or telephone pole on your block	1	2	3	4	5
e. Having mosquitoes outside your house	1	2	3	4	5
f. Getting seven mosquito bites in a week	1	2	3	4	5

17. I want you to think of the following yard and porch activities (**NOTE: READ THE LIST)**. How much do you enjoy participating in these activities in your yard or around your home? Please rate them from 1 to 5. Where 1= enjoy them a great deal and 5= do not enjoy at all. (**NOTE: HAND CARD Q17 TO INTERVIEWEE AND GIVE TIME TO THINK**)

→

Least enjoyable (1) **Most enjoyable (5)**

a. Eating or cooking outside	1	2	3	4	5
b. Playing catch, Frisbee, bocce, horseshoes, croquet, volleyball, etc.	1	2	3	4	5
c. Relaxing, socializing, talking, reading, hanging out etc.	1	2	3	4	5
d. Gardening or mowing lawn	1	2	3	4	5
e. Working on or repairing your house or car	1	2	3	4	5

NOTE: USE ANSWER SHEET TO RECORD THE ANSWERS FOR Q 18 TO 27 (RESPONSE TABLE 1 AND RESPONSE FIGURE 1)

18. Now, I will ask you a few questions about an imaginary hour and how you would use it. I want you to imagine that you have **one additional work-free hour each summer week** to spend doing yard and porch activities. This could occur if you got out of work or school an hour earlier, the traffic or buses were faster, or you finished your household tasks sooner. This hour would be free of responsibilities and available only for your pleasure and enjoyment. Furthermore, I want you to think of it as a **one-mosquito-free yard and porch hour**. If you could spend this hour doing just one of the following activities, which one would you choose?
    a. Eating or cooking outside
    b. Playing catch, frisbee, Bocce, horseshoes, croquet, volleyball, etc.
    c. Relaxing, socializing, talking, reading, hanging out, etc.
    d. Gardening or mowing lawn
    e. Maintaining house or car

Now I would like to know how much this additional hour is worth for you. By having a value for this hour we will be able to understand your preferences and help policy makers develop policies that address these preferences.

19. Would you be willing to pay $1 to have one additional hour for DOING ACTIVITY X? (Use Figure 1 as a guide)

20. Now, I want you to think again of question Q18, and imagine the same one additional work-free hour that you can spend on the following remaining 4 activities. Which activity will you choose to spend this hour on?

21. Would you be willing to pay $1 to have one additional hour for DOING ACTIVITY X? (Use Figure 1 as a guide)

22. Now, I want you to think again of Q18, and imagine the same one additional work-free hour that you can spend on the remaining 3 activities, which activity will you choose to spend this hour on?

23. Would you be willing to pay $1 to have one additional hour for DOING activity X? (Use Figure 1 as a guide)

24. Now, I want you to think again of question Q18 and imagine the same one additional work-free hour that you can spend on the remaining 2 activities, which activity will you choose to spend this hour on?

25. Would you be willing to pay $1 to have one additional hour for DOING activity X? (Use Figure 1 as a guide)

25a. Would you be willing to pay $1 to have one additional hour for DOING activity X? (Use Figure 1 as a guide)

RESPONSES TABLE 1 for questions 18-25

Activity	Ranking of activities (Q18,20,22 and 24)	Would you be willing to pay $1 to have one additional hour for DOING ACTIVITY (Maximum amount respondent willing to pay) (Q19, 21, 23,25 and 25a)
a. Eating and cooking outside		
b. Playing catch, frisbee, Bocce, horseshoes, croquet, volleyball etc.		
c. Relaxing, socializing, talking, reading, hanging out etc.		
d. Gardening and mowing lawn		
e. Working on or repairing your house or car		

RESPONSE FIGURE 1 for questions 18-25a

Figure 1. Willingness to pay chart

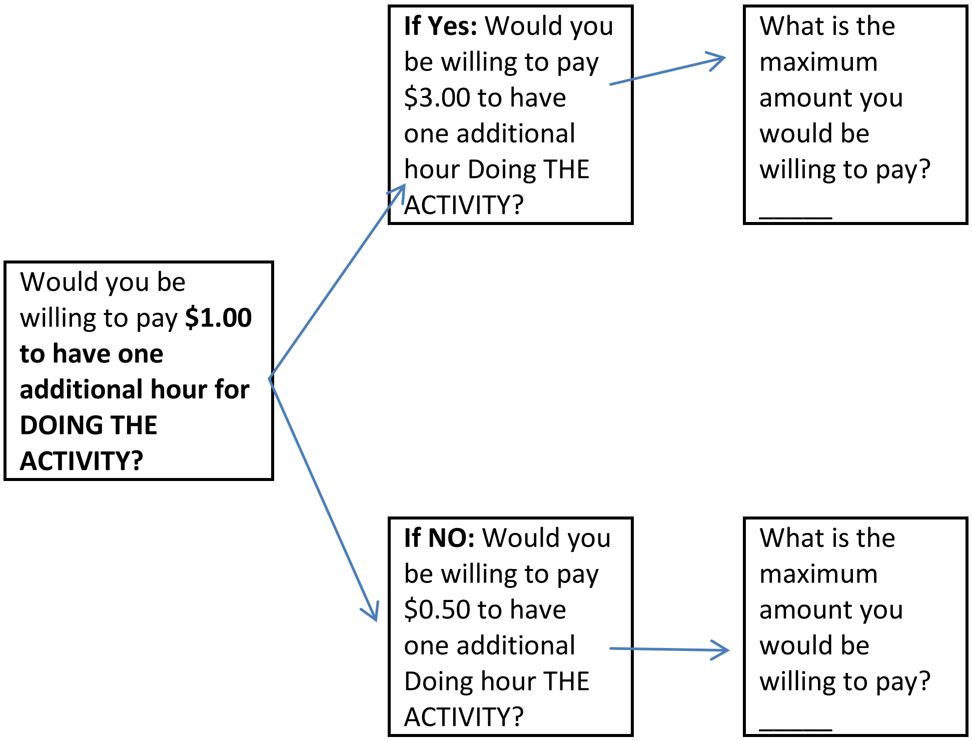

Quality of life question:

I want you think of your yard or porch. If you don’t have a yard or porch, I want you to think of or imagine one that you have access to and you can use anytime you want. Now I want you to imagine the following best and worst scenarios:

No mosquitoes present (best scenario). There is no mosquito problem. You are able to do everything you want in your yard and porch at any time with no restrictions.

Mosquito invasion (worst scenario). Mosquitoes infested your yard and porch; they are getting into your house and they are uncontrollable. Spending 10 minutes in your yard or porch will result in 10 bites across your body, including 2 on your face, leading to very itchy or painful welts. You and your household will be forced to cancel your yard and porch activities. Mosquitoes will limit your social activities with neighbors and community, and stop you from exercising outdoors. Fear of mosquitoes and the need to stay indoors will make you anxious. Continued use of bug spray and other chemicals will smell bad and may stain your clothes.

Now, I want you to think of your experience with mosquitoes during this summer and place **your current mosquito experience on a scale from 100 referring to no mosquitoes (the best scenario) to 0 referring to the mosquito invasion (worst scenario)** mentioned above.

(**NOTE: HAND CHART Q26 TO INTERVIEWEE AND GIVE TIME TO THINK**)

26. Now I want you to think of the current level of mosquitoes around your house and compare it to the following states and chose which is worst:

(NOTE: READ FROM CARDSQ27.2-5 AND RECORD THE ANSWER BY CIRCLING THE WORST CASE REPORTED BY INTERVIEWEE)

1	a	Living an average day with **some problems with performing your usual activities** (i.e. work, study, housework, family or leisure activities), but no problems in walking about, no problem with self-care, no pain or discomfort, and not anxious or depressed (11211)	b	Living an average day with mosquitoes as they were this past summer in your yard and porch
2	a	Living an average day with **some problems walking about**, but no trouble washing or dressing yourself, having no trouble with self-care, no problems performing your usual activities (i.e., work, study, housework, family or leisure activities), no pain or discomfort, not anxious or depressed (21111)	b	Living an average day with mosquitoes as they were this past summer in your yard and porch
3	a	Living an average day with **moderately anxious or depressed**, but no problem in walking about, no problem with self-care, no problems performing your usual activities (i.e. work, study, housework, family or leisure activities), no pain or discomfort, (11112)	b	Living an average day with mosquitoes as they were this past summer in your yard and porch
4	a	Living an average day with **some problems in walking about, some problems with self-care**, but no problems in performing your usual activities (i.e. work, study, housework, family or leisure activities), no pain or discomfort and not anxious or depressed (22111)	b	Living an average day with mosquitoes as they were this past summer in your yard and porch

5	a	Living an average day with **some problems with self-care, modest anxious or depression,** but no trouble walking about, no problems performing your usual activities (i.e. work, study, housework, family or leisure activities), no pain or discomfort, (12112)	b	Living an average day with mosquitoes as they were this past summer in your yard and porch

27. Now I want you to think of the following conditions and choose the worst option:
(NOTE: READ FROM CARDS Q28 1-5 AND RECORD THE ANSWER BY CIRCLING THE WORST CASE REPORTED BY INTERVIEWEE)

1	a	Living an average day with influenza	b	Living an average day with mosquitoes as they were this past summer in your yard and porch
2	a	Living an average day with a stomach flu (severe diarrhea and vomiting)	b	Living an average day with mosquitoes as they were this past summer in your yard and porch
3	a	Living an average day with severe hearing loss	b	Living an average day with mosquitoes as they were this past summer in your yard and porch
4	a	Living an average day with a wrist fracture	b	Living an average day with mosquitoes as they were this past summer in your yard and porch
5	a	Living an average day with a bronchitis	b	Living an average day with mosquitoes as they were this past summer in your yard and porch

28. Apart from this or any previous survey, had you ever seen or heard of a black and white striped mosquito called the Asian tiger mosquito, considered a potential carrier of diseases?
    a. Yes
    b. No

29. Has your selected child discussed the Asian tiger mosquito with you?
    a. Yes
    b. No

30. Did your selected child learn about mosquitoes in his/her elementary school?
    a. Yes
    b. No

31. Have you, or your selected child, done any of the following actions around your house and/or yard this summer?

Action	You			Selected child		
	Yes	No	NA*	Yes	No	NA*
a. Cleaned gutters						
b. Placed insecticides (such as mosquito dunks®) into standing water						
c. Emptied water out of outdoor containers						
d. Placed fish into containers outdoors						
e. Stored containers upside down						
f. Requested visits from pest control company						
g. Checked backyard weekly for standing water						
h. Drilled holes into trashcans						
i. Removed tires from backyard						

*NA= Not applicable (for example, you don't have a yard; the situation did not exist at the beginning of the summer; the action is not your responsibility)

32. Do you have plastic toys, pet watering dishes, and/or plant saucers outside your house?
    a. Yes
    b. No
    c. Not applicable or don't know

33. Is it possible for mosquitoes to hatch in water allowed to collect in plastic toys left outdoors?
    a. Yes
    b. No
    c. Don't know

34. In general, Standing water +______ days = Mosquitoes? (**NOTE: MARK ONE**)
______ 3 days
______ 7 days
______ 14 days
______ 21 days
______ Don't know

35. Which of the following statements is true:
______ Only the male mosquito can bite
______ Only the female mosquito can bite
______ Both the male and female mosquito can bite
______ Don't know

36. **During this summer**, how much did your household pay for the following items specifically to control mosquitoes? **(NOTE: IF NONE, PUT 0)**

$_____________ Repairing leaky outdoor faucets and/or water pipes
$_____________ Repairing window and/or door screens
$_____________ Adding insecticides to standing water outside your house
$_____________ Bug zapper, mosquito trap, light magnet, mosquito magnet, propane trap
$_____________ Spray to repel mosquitoes
$_____________ Burning coils or candles to repel mosquitoes
$_____________ Other, specify__________________________

37. Over the last week, did you and/or your selected child use insect repellent, such as Cutter ®, during outdoor porch or yard activities as protection against mosquitoes? (**NOTE: MARK ONE)**

**Response**	**You**	**Selected child**
Yes		
Sometimes		
No		

38. How many people live in your household (**including** yourself)?
____

39. How many people in your household (**including** yourself) are in the following age groups?

__________ Under 15 years
__________ 15-24
__________ 25-34
__________ 35-44
__________ 45-54
__________ 55-64
_________ 65-74
_________ 75-84
_________ over 85

40. Of the total number of children in your household, how many are currently enrolled in elementary school? _______________

41. What is the gender of your selected child?
   a. Boy
   b. Girl

42. Which school does your selected child attend?
____

43. What is the level of your selected child's school?
_____ Elementary
_____ Junior high school or middle school
_____ High school
_____ Post-secondary institution (e.g. college)
_____ Others

44. In which age group are you?

__________ 15-24
__________ 25-34
__________ 35-44
__________ 45-54
_________ 55-64
_________ 65-74
_________ 75-84
_________ over 85

45. What is your gender? **(NOTE: MARK ONE)**

a. Male
b. Female

46. What is your level of education? (**NOTE: MARK ONE**)
_____ Less than 9th grade
_____ 9th to 12th grade (no diploma)
_____ High school graduate or equivalent
_____ Some college, no degree
_____ Associate's degree
_____ Bachelor's degree
_____ Graduate or professional degree

47. What is your current employment status? **(NOTE: MARK ONE)**
_____ Employed full time
_____ Employed part time
_____ Active at home (homemaker, parent)
_____ Student, not working
_____ Unemployed - looking for a job
_____ Not working - not looking for a job
_____ Retired

THANK YOU FOR YOUR PARTICIPATION.
